# Ophthalmological Society of the United Kingdom

**Published:** 1888-01

**Authors:** 


### OPHTHALMOLOGICAL SOCIETY OF THE UNITED KINGDOM.

LONDON, Thursday, Dec. 8, 1887.

J. W. Hulke, F.R.C.S., F.R.S., President, in the Chair.

*An Unusual Complication after Subconjunctival Tenotomy of the External Rectus.*—Dr. A. Emrys-Jones, Manchester, read this paper, relating to a little girl aged 10, in whom both internal recti were divided for alternating convergent strabismus. Immediately after division of the left rectus there was profuse hæmorrhage into Tenon's capsule, leading to well-marked proptosis. A firm bandage was applied, but two hours later the proptosis was more marked, the pupil was widely dilated, inactive to light, and perception of light very doubtful. The optic disc was very pale. From this time rapid improvement took place, and recovery was eventually complete.

*Persistent Hæmorrhage in the Anterior Chamber after Iridectomy for Chronic Glaucomy.*—Dr. Emrys-Jones also read this case: The patient was a man aged 63, whose right eye was almost blind, and with + 1 tension; in the left eye, vision $\frac{6}{18}$, and the field of vision much contracted. The optic nerve was cupped. Iridectomy was performed on October 24, 1882. Free hæmorrhage followed, and the next day the anterior chamber was full of blood, and no trace of iris could be detected anywhere. There was scarcely any improvement in a fortnight. One month after operation the hæmorrhage had subsided considerably, and the outline of the pupil could be detected. The improvement was excessively slow, and it was not until December 16th that an ophthalmoscopic examination could be made, when the vitreous was found to be clear and the optic nerve much cupped. There was cardiac disease, and the patient died on January 17, 1883, from double pneumonia. Mr. Haynes Walton had written something about this condition in his book when treating of injuries to the eye from mechanical causes.

The President asked what method of subconjunctival tenotomy had been adopted in the first case; he could not recall any similar occurrence. In the second patient the case was one of persistent hæmorrhage, the blood retaining its color, as was sometimes seen in diabetic patients.

Mr. Adams Frost and Mr. Ernest Clarke had met with cases exactly like the one recorded in the first paper, the latter speaker suggesting that division of the tendon too near its insertion was, perhaps, the cause.

The President pointed out that the object of Von Graefe's operation was to divide the tendon as close to the globe as possible.

Mr. Lawford had seen one case in which severe hæmorrhage had followed tenotomy, leading to proptosis and duskiness of the lips. The patient, a boy, went on well at first, but subsequently had a relapse after a sudden effort.

Mr. Brailey had seen instances of this accident, in one case leading to atrophy of the optic nerve. He had always been inclined to attribute the occurrence to a too free use of the hook rupturing one of the ciliary arteries, and he considered Von Graefe's operation very unlikely to lead to such an accident.

*On Congenital and Hereditary Defect of Ocular Movements.*—Mr. Lawford read notes of four cases which had been under his care at St. Thomas' Hospital. These cases included a father and three of his seven children, the remaining four being unaffected. The three children who were the subjects of this congenital defect were numbers 2, 4, and 7. In all four patients there were present the following symptoms: Almost complete bilateral ptosis, loss of upward and downward movement of the eyeballs, and very defective lateral movement. Visual acuteness and accommodative power were good, and there were no ophthalmoscopic changes. None of the patients presented any other congenital defect, and they were healthy in every other respect. He referred to published records of similar cases, in some of which *post-mortem* examinations had been obtained, and drew attention to the fact that the ocular muscles, and not their nerves, were the structures at fault. These muscles had, in some instances, been absent; in others, though present, they were occasionally ill-developed, and generally shorter than the average measurement, while in almost every case their insertions into the sclerotic were displaced more or less backwards, the greatest degree of displacement being 2.5 mm.

The President said that it was difficult to say whether the muscles were primarily at fault, or whether there was defective innervation of muscles; disease in early life might produce such effects.

Mr. Quarry Silcock supported the last idea, and related the case of a boy in whom the eyeball was not noticed to be fixed till the age of three years, and after a general illness which might have been infantile palsy, with a localization in the eyeball muscles.

Mr. Henry Power spoke of the solidarity between nerve and muscle; the condition might be looked upon in some cases as a reversion to an earlier type; turtles and other reptiles had little movement, and perhaps some mammals might show an absence of the levator.

Dr. Angel Money objected to the principle of so-called reversion to an earlier type, and preferred to speak of an arrest of development.

Mr. Brailey said that the development of the ocular muscles was not known; he did not think the reversion theory would hold good in the present instance.

Dr. Seymour Sharkey and Mr. Doyne thought that the term reversion was a useful one.

*Living and Card Specimens.*—Mr. Silcock showed a case of exostosis arising from the inner side of the orbit; sight had been early affected and was almost completely lost.

Mr. Hulke thought an exploration should be made; in one case in his own experience the tumor proved easy of removal.

Mr. Henry Power had had considerable difficulty in removing an exostosis from the roof of the orbit.

Mr. Nettleship, in reply to Mr. Silcock, said the sight failed after the proptosis had been present one year.

Mr. Spencer Watson considered exploration would be necessary; but caution should be used not to employ too much force in chiselling, etc.

Mr. Gunn: A peculiar foveal reflex in connection with amblyopia and myopia.

Mr. Nettleship showed an ophthalmoscopic drawing from a case of night blindness, with white dots scattered over the fundus. A discussion followed relative to whether these spots were identical with those seen in Tay's choroiditis.

